# Ammonia Excretion in an Osmoregulatory Syncytium Is Facilitated by AeAmt2, a Novel Ammonia Transporter in *Aedes aegypti* Larvae

**DOI:** 10.3389/fphys.2018.00339

**Published:** 2018-04-11

**Authors:** Andrea C. Durant, Andrew Donini

**Affiliations:** Department of Biology, York University, Toronto, ON, Canada

**Keywords:** ammonia transporters, Rhesus glycoproteins, mosquito, high environmental ammonia (HEA), anal papillae

## Abstract

The larvae of the mosquito *Aedes aegypti* inhabit ammonia rich septic tanks in tropical regions of the world that make extensive use of these systems, explaining the prevalence of disease during dry seasons. Since ammonia (NH_3_/${\text{NH}}_{4}^{+}$) is toxic to animals, an understanding of the physiological mechanisms of ammonia excretion permitting the survival of *A. aegypti* larvae in high ammonia environments is important. We have characterized a novel ammonia transporter, *AeAmt2*, belonging to the Amt/MEP/Rh family of ammonia transporters. Based on the amino acid sequence, the predicted topology of AeAmt2 consists of 11 transmembrane helices with an extracellular N-terminus and a cytoplasmic C-terminus region. Alignment of the predicted AeAmt2 amino acid sequence with other Amt/MEP proteins from plants, bacteria, and yeast highlights the presence of conserved residues characteristic of ammonia conducting channels in this protein. AeAmt2 is expressed in the ionoregulatory anal papillae of *A. aegypti* larvae where it is localized to the apical membrane of the epithelium. dsRNA-mediated knockdown of AeAmt2 results in a significant decrease in ${\text{NH}}_{4}^{+}$ efflux from the anal papillae, suggesting a key role in facilitating ammonia excretion. The effect of high environmental ammonia (HEA) on expression of AeAmt2, along with previously characterized AeAmt1, AeRh50-1, and AeRh50-2 in the anal papillae was investigated. We show that changes in expression of ammonia transporters occur in response to acute and chronic exposure to HEA, which reflects the importance of these transporters in the physiology of life in high ammonia habitats.

## Introduction

Mosquito larvae (order Diptera) are found in a wide range of habitats which vary in alkalinity and ionic composition. The disease vector mosquito, *Aedes aegypti*, is historically considered a fresh water breeder where the larvae are commonly found in clean freshwater habitats including artificial containers, streams, and pools (Ramasamy et al., 2011). Few mosquito species are capable of surviving in heavily polluted waters, *A. aegypti* being one such species. In fact, there is a growing body of evidence from field studies in tropical regions of the world demonstrating that *A. aegypti* are abundant in septic tanks containing raw sewage (Barrera et al., 2008; Burke et al., 2010; Banerjee et al., 2015; Chitolina et al., 2016). In some regions, it was estimated that these cryptic aquatic environments were providing a new habitat for more than 18,000 emerging *A. aegypti* adults daily (Barrera et al., 2008). A number of observations suggest that *A. aegypti* may be well adapted to inhabiting sewage. When female *A. aegypti* were given a choice to lay their eggs in freshwater or raw sewage, there was no breeding preference displayed (Chitolina et al., 2016). Furthermore, *A. aegypti* emerging from septic tanks have longer wings and higher nutrient reserves than those emerging from freshwater (Banerjee et al., 2015). This is significant as these are positive measures of overall fitness because larval nutrient acquisition is an important determinant of successful breeding of adults. While no genetic differentiation was observed between *A. aegypti* populations from septic tanks and freshwater surface containers, it was suggested that septic tank populations may even represent a more dangerous phenotype for disease transmission (Somers et al., 2011).

Sewage contains high levels of ammonia (NH_3_/${\text{NH}}_{4}^{+}$) which is toxic to animal cells at micromolar concentrations (Weihrauch et al., 2004). Furthermore, free ammonia and ammonium salts are common in organically polluted water inhabited by these mosquitoes (Kell Reid, 1961; Mitchell and Wood, 1984). *A. aegypti* larvae are tolerant to remarkably high levels of ammonium chloride (~7 mM NH_4_Cl), the major toxic component of sewage, and selection toward an increased tolerance to high ammonia was observed within a single generation (Mitchell and Wood, 1984). This is unlike other aquatic invertebrate species where levels as low as 58 μmol l^−1^ of NH_3_ and 1.39 mmol l^−1^ total ammonia is lethal (Chen and Lin, 1992; Wright, 1995; Weihrauch et al., 2004; Weihrauch and Donnell, 2017). These findings raise the question of what physiological mechanisms *A. aegypti* larvae possess allowing for high ammonia tolerance. Ammonia transport and excretion is a fundamental physiological process, one that all animals possess in some capacity (Marini et al., 1994, 1997, 2000). In *A. aegypti*, the anal papillae have been shown to excrete ammonia and research on the molecular mechanisms of ammonia excretion by these organs has recently begun (Chasiotis et al., 2016; Durant et al., 2017).

The four anal papillae in *A. aegypti* larvae are relatively elongate sac-like structures that surround the anal opening and are the product of eversion of the hindgut tissues (Edwards and Harrison, 1983). Anal papillae are composed of a single layer of homogenous cells that form a syncytium covered by a thin cuticle. The apical surface of the epithelial cells is directed outwards and the basal membrane faces the papilla lumen. The lumen of the papillae contains hemolymph and is continuous with the hemocoel of the body (Sohal and Copeland, 1966). In *A. aegypti*, the anal papillae are important for ionoregulation whereby active uptake of ions (Na^+^, Cl^−^) from the surrounding dilute medium occurs in addition to ammonia excretion (Donini and O'Donnell, 2005).

Ammonia transport across biological membranes is facilitated by Methylammonium/ammonium permeases (MEP) in yeast, also known as ammonium transporters (Amt) in bacteria, as well as analogs of both in vertebrates, the conserved family of Rhesus glycoproteins (Rh proteins) (Kustu and Inwood, 2006). Invertebrates possess both Amt/MEP and Rh proteins (Gruswitz et al., 2010). The Rh proteins are glycosylated, comprising a group of Rh-50 proteins (~50 kDa) which function as trimers (Gruswitz et al., 2010). Each monomer possesses 12 transmembrane helices, one more than invertebrate, plant, and bacterial homologs, which form a triple pore for substrate transfer through each monomer comprising the trimer. The crystallographic structure of mammalian RhCG predicts the transport of NH_3_ over ${\text{NH}}_{4}^{+}$, whereby RhCG recruits ${\text{NH}}_{4}^{+}$ which is then deprotonated and NH_3_ is conducted through the channel (Kustu and Inwood, 2006; Gruswitz et al., 2010; Baday et al., 2015). The proton is recycled back to the extracellular space, resulting in electroneutral transport through these passive gas channels (Li et al., 2007; Lupo et al., 2007; Weihrauch and O'Donnell, 2015). Rh proteins are also proposed to be CO_2_ gas channels in addition to ammonia transporters and require a partial pressure gradient (Δ*P*_NH3_ and Δ*P*_CO2_) for transport (Khademi et al., 2004). Amt/MEP proteins have 11 pore-forming transmembrane helices which trimerize in the membrane, forming a triple pore for ammonia transport similarly to Rh proteins (Khademi et al., 2004; Zheng et al., 2004). The crystal structure of AmtB in *Escherichia coli* indicates that the trimer has a hydrophobic pore located at the center of each monomer with an NH_3_/${\text{NH}}_{4}^{+}$ binding site at the entry of each pore, and uncharged NH_3_ is transported through the channel (Mayer et al., 2006). Some plant AMTs such as the *Lycopersicon esculentum* LeAMT1;2, are electrogenic ammonia transporters which conduct ${\text{NH}}_{4}^{+}$ specifically, or act as a cotransporter for NH_3_/H^+^ (Ludewig et al., 2002; Mayer et al., 2006). The anal papillae epithelium is a suitable tissue for electrogenic transport of ${\text{NH}}_{4}^{+}$, because the transport activities of basolateral Na^+^/K^+^-ATPase (NKA) and apical V-type H^+^-ATPase (VA) is expected to produce a large cytosol negative voltage potential which would favor ${\text{NH}}_{4}^{+}$ entry into the cytosol from the hemolymph (Patrick et al., 2006; Weihrauch et al., 2012b; Chasiotis et al., 2016).

Amt/MEP and Rh proteins have been identified and characterized as ammonia transporters in anopheline and aedine mosquito species (Wu et al., 2010; Pitts et al., 2014). We have previously identified and characterized one Amt/MEP, *AeAmt1*, and two Rh proteins, *AeRh50-1* and *AeRh50-2*, in *A. aegypti* larvae which are expressed in the ionoregulatory syncytial epithelium of the anal papillae and have been implicated in facilitating ammonia excretion within this organ (Chasiotis et al., 2016; Durant et al., 2017). Double-stranded RNA (dsRNA)-mediated knockdown of either *AeAmt1, AeRh50-1*, or *AeRh50-2* within the anal papillae of *A. aegypti* larvae causes a significant decrease in ${\text{NH}}_{4}^{+}$ efflux from the papillae and altered ${\text{NH}}_{4}^{+}$ and pH levels in the hemolymph.

This suggests that each of these transporters plays a role in ammonia excretion at the anal papillae at the very least (Chasiotis et al., 2016; Durant et al., 2017). Changes in Rh protein expression in leeches, crabs, frogs, and fish in response to high environmental ammonia (HEA) has been well documented (Hung et al., 2007; Martin et al., 2011; Cruz et al., 2013; Quijada-Rodriguez et al., 2015), however, the involvement of Amts in invertebrates and Rh proteins in mosquitoes, specifically, in response to HEA is unknown.

The aim of this study was to characterize a novel Amt protein in the anal papillae of *A. aegypti* larvae and examine changes in ammonia transporter expression in response to HEA. We hypothesized that the novel Amt protein, AeAmt2 is expressed in the anal papillae where it functions to facilitate ammonia excretion, and that HEA exposure will result in alterations of ammonia transporter expression. To test this hypothesis, we examined transcript and protein expression of *AeAmt2* and conducted functional studies using a combination of dsRNA with electrophysiology techniques. Furthermore ammonia transporter expression in response to HEA was examined.

## Materials and methods

### Animals and rearing conditions

A colony of *Aedes aegypti* (Linnaeus) was established in 2007 at York University with eggs obtained from Dr. Marjorie Patrick at the University of San Diego, CA, USA. The colony has been supplemented with eggs from a colony of *Aedes aegypti* (Liverpool strain) at Simon Fraser University, B.C., Canada in the laboratory facilities of Dr. Carl Lowenberger. The procedures for rearing mosquitoes were adapted from a previously established protocol (Donini and O'Donnell, 2005). Cages containing adult mosquitoes were kept in the laboratory at room temperature and had continuous access to a 5% aqueous sucrose solution. Females were fed on warm sheep's blood in Alsever's solution (Cedarlane Laboratories, Burlington, ON. Canada). Eggs were collected on filter paper and stored dry in plastic containers until needed. Eggs were hatched and larvae reared in dechlorinated tap water (deH_2_O) in plastic containers in the laboratory. Lights on a timer located at the bench where the mosquito cages and larval containers were held simulated a 12:12 light:dark cycle. Larvae were fed daily with a solution of liver powder and yeast in water. Rearing water was refreshed every other day. Fourth instar larvae were used 24 h post-feeding for physiological and molecular studies.

### Animals and rearing conditions for high environmental ammonia (HEA) studies

HEA treatments conducted in this study were 2-fold; (1) acute (6 h) and semi-chronic (48 h) exposure to HEA, and (2) rearing (7 days) in HEA. For acute (6 h) and semi-chronic (48 h) exposure to HEA studies, larvae were hatched and reared in deH_2_O until reaching fourth instar when they were then transferred to either deH_2_O or 5 mM NH_4_Cl in deH_2_O for 6 and 48 h. For rearing in HEA experiments, larvae were hatched in deH_2_O and transferred to either deH_2_O or 5 mM NH_4_Cl in deH_2_O 2 days post hatching until they reached fourth instar.

### Reverse-transcriptase PCR (RT-PCR) and quantitative real-time PCR (qRT-PCR)

AeAmt2 was identified by sequence alignment to the AeAmt1 nucleotide sequence using a BLAST query from the Bioinformatics Resource for Invertebrate Vectors of Human Pathogens (VectorBase) and the nucleotide database provided by the National Center for Biotechnology Information (NCBI). Partial mRNA sequences for *A. aegypti AeAmt2* (AAEL007373-RA) were used for primer design (forward primer: 5′-GCATTTTAGCGTCACTGGTC-3′; reverse primer: 5′-GGGAATAGGGTTATCAGCAAAC-3′; 221 bp amplicon size, 62°C annealing temperature). Primers for *AeAmt1, AeRh50-1*, and *AeRh50-2* were previously designed and described (Chasiotis et al., 2016). Three biological samples, each consisting of a pool of 200 anal papillae from 50 larvae were isolated and collected in cold lysis buffer with 1% 2-mercaptoethanol (Ambion, Austin, TX, USA). Total anal papillae RNA was extracted using the Purelink RNA mini kit (Ambion, Austin, TX) and was treated with the TURBO DNA-free™ Kit (Applied Biosystems, Streetsville, Ont, Canada) to remove genomic DNA. Template cDNAs were synthesized using the iScript™ synthesis kit (Bio-Rad, Mississauga, ON, Canada) with 1 μg of total RNA for each reaction. PCR amplicons of *AeAmt2, AeRh50-1, AeRh50-2, AeAmt1* were resolved by agarose gel electrophoresis with ethidium bromide. AeAmt2 transcript was concentrated and purified using the QIAquick PCR Purification Kit (Qiagen, Toronto, Ont, Canada), and was sequenced (Bio Basic Inc., Markham, ON, Canada) to ensure the specificity of the product.

Quantitative real time PCR (qRT-PCR) using the primers described above was used to examine the mRNA abundance of *AeRh50-1, AeRh50-2, AeAmt1*, and *AeAmt2* in the anal papillae. qRT-PCR reactions were carried out using the CFX96™ real-time PCR detection system (Bio-Rad) and SsoFast™ Evagreen® Supermix (Bio-Rad) according to the manufacturer's protocol. Ribosomal 18s RNA served as the reference gene utilizing primers that have been previously reported (Sanders et al., 2003; Chasiotis et al., 2016; Jonusaite et al., 2016). A melting curve analysis was performed after each cycle to confirm the presence of a single product. For *AeAmt2* primer optimization, a standard curve was generated to assess primer and reaction efficiency. Quantification of relative transcript abundance was determined according to the Pfaffl method (Pfaffl, 2004). For the expression profile of Amt and Rh proteins in the anal papillae, the mRNA abundance of ammonia transporter genes in the anal papillae were expressed relative to *AeRh50-1*, which was assigned a value of 1.0 after normalizing with 18s RNA abundance. To assess effects of HEA treatment on mRNA abundance of ammonia transporter genes the mRNA abundance of each respective gene was expressed relative to the control deH_2_O, which was assigned a value of 1.0 after normalizing to 18s transcript abundance.

### Western blotting and immunohistochemistry

The protein abundance of AeAmt2, AeAmt1, and AeRh50s in the anal papillae of 4th instar larvae was examined using Western blotting. Briefly, biological samples consisting of pooled anal papillae were isolated from 20 to 50 larvae in *A. aegypti* saline (Donini et al., 2006) and were sonicated (3 × 10 s) in a homogenization buffer (50 mmol l^−1^ Tris-HCl pH 7.4, 1 mmol l^−1^ PMSF, 150 mmol l^−1^ NaCl, 1% sodium deoxycholate, 1% Triton X-100, 0.1% SDS, and 1:200 protease inhibitor cocktail [Sigma-Aldrich]), centrifuged at 13,000 g for 10 min at 4°C, and the supernatant was stored at −80°C. Samples consisting of 5 μg of protein, determined using the Bradford assay (Bio-Rad), were prepared for SDS-PAGE by heating for 5 min at 100°C in 6 × loading buffer (360 mmol l^−1^ Tris-HCl pH 6.8, 12% (w/v) SDS, 30% glycerol, 600 mmol l^−1^ DTT, and 0.03% (w/v) Bromophenol blue) and then were electrophoretically separated by SDS-PAGE (12% polyacrylamide). Western blot analysis of each ammonia transporter was conducted according to an established protocol (Chasiotis and Kelly, 2008; Chasiotis et al., 2016; Durant et al., 2017). A custom-synthesized polyclonal antibody that was raised in rabbit against a synthetic peptide corresponding to a 14-amino acid region (DKMSPQKKANDQPK) of *AeAmt2* (GenScript USA Inc., Piscataway, New Jersey, USA) was used at a 1:5,000 dilution. Custom-synthesized polyclonal antibodies raised in rabbit against AeAmt1 and AeRh50s were used at dilutions of 1:500 and 1:2,000, respectively, and have been previously validated (Chasiotis et al., 2016; Durant et al., 2017). The AeRh50 antisera is presumed to detect both AeRh50-1 and AeRh50-2 (Durant et al., 2017). AeAmt2 antibody specificity was confirmed by running a comparison blot with AeAmt2 antibody pre-absorbed with 10× molar excess of immunogenic peptide for 1 h at room temperature prior to application to blots. After examination of ammonia transporter expression, blots were stripped and either re-probed with a 1:1,000 dilution of rabbit monoclonal anti-GAPDH antibody (14C10, New England BioLabs, Whitby, ON, Canada) following the above procedure, as specified, or total protein analysis was carried out using Coomassie total protein staining as a loading control (0.1% Coomassie R250, 50% methanol, 50% ddH_2_O) (Eaton et al., 2013). Preliminary studies were used to determine which loading control was appropriate for the study treatments. For the 6 and 48 h HEA exposures, GAPDH expression did not change with treatment and was therefore used as the loading control. For dsRNA and 7 day HEA treatments, GAPDH expression increased and therefore total protein was used as the loading control. Blots were incubated in Coomassie for 1 min followed by de-staining for 3–5 min in de-stain solution (50% ethanol, 10% acetic acid, 40% ddH_2_O). Blots were then washed in ddH_2_O for 1 min and dried completely prior to visualization using a Gel Doc XR + system (Bio-Rad). Densitometric analysis of AeAmt2, AeAmt1, AeRh50s, GAPDH, and Coomassie total protein was conducted using ImageJ 1.50i software (National Institutes of Health, Bethesda, MD, USA).

Immunolocalization of AeAmt2 in paraffin-embedded cross sections of anal papillae was conducted using immunohistochemistry. Na^+^-K^+^-ATPase (NKA) and the V_0_ subunit of V-type H^+^ ATPase (VA) immunostaining were used as markers for the basolateral membrane and apical membrane, respectively (Patrick et al., 2006). The procedure was carried out according to an established protocol (Chasiotis et al., 2016; Durant et al., 2017). AeAmt2 antibody was used at a 1:500 dilution, a mouse monoclonal anti-α5 antibody for NKA (Douglas Fambrough, Developmental Studies Hybridoma Bank, IA, USA) was used at a concentration of 4.2 ug/mL (Patrick et al., 2006; Chasiotis et al., 2016; Durant et al., 2017), and a guinea pig anti-V-type H^+^-ATPase (kind gift from Dr. Weiczorek, University of Osnabruk, Germany) was used at a 1:3,000 dilution. A sheep anti-mouse antibody conjugated to Cy2 and a goat anti-guinea pig antibody conjugated to AlexaFluor 647 (Jackson ImmunoResearch Laboratories, West Grove, PA, USA) at dilutions of 1:500 was used to visualize NKA and VA, respectively. A goat anti-rabbit antibody conjugated to Alexa Fluor 594 (Jackson ImmunoResearch) at a dilution of 1:500 was applied to visualize AeAmt2. Comparison control slides were also processed as described above, with control slides incubated in primary immune serum with no primary antibody. Slides were mounted using ProLong Gold antifade reagent with DAPI (Life Technologies, Burlington, ON). Fluorescence images were captured on a Zeiss LSM 700 laser scanning microscope (Zeiss, U.S.A). Images were merged using ImageJ 1.50i software (National Institutes of Health, Bethesda, MD, USA).

### dsRNA synthesis and delivery

Using the NCBI nucleotide database, primers were designed for *AeAmt2* dsRNA synthesis that spans 951 bp of the partial mRNA sequence (forward: 5′-ACTCAGGCAGCACATACGG-3′; reverse: 5′-ATTGATTTCCCCCCAACTCG-3′). The purified PCR product was sequenced (The Centre for Applied Genomics, Sick Kids Hospital, Toronto, ON) to ensure specificity of the target. A fragment of the β*-lactamase* (β*-lac*) gene was also amplified by RT-PCR from a pGEM-T-Easy vector (kind gift from Jean-Paul Paluzzi) (forward: 5′-ATTTCCGTGTCGCCCTTATTC-3′; reverse: 5′-CGTTCATCCATAGTTGCCTGAC-3′, 799 bp amplicon size) and served as a control. Using the PCR product as template, RT-PCR was carried out using the *AeAmt2* and β*-lac* primers with an additional T7 promoter sequence (5′-TAATACGACTCACTATAGGG-3′). *AeAmt2* and β*-lac* PCR products with the T7 promoter were concentrated and purified using the QIAquick PCR Purification Kit (Qiagen). dsRNA was synthesized according to a previously described protocol (Chasiotis et al., 2016), using the Promega T7 RiboMAX Express RNAi kit (Promega, Madison, WI, USA) and the *AeAmt2* and β*-lac* T7 PCR products as template.

For *AeAmt2* dsRNA-mediated knockdown studies, 30 fourth instar larvae were incubated in 0.5 μg μl^−1^ dsRNA in 150 μl PCR-grade water for 2 h (Singh et al., 2013), after which they were transferred into 50 ml of deH_2_O. Larvae ingest the media that they inhabit as shown in previous studies (Singh et al., 2013; Chasiotis et al., 2016). Water was refreshed every other day and larvae were fed every other day beginning 24 h after dsRNA treatment.

### Ion-selective microelectrodes (ISME) for hemolymph ion measurements

For hemolymph collection, larvae were gently blotted on filter paper to remove excess water. Larvae were then submerged in paraffin oil (Sigma-Aldrich, Oakville, ON, CA) and fine forceps were used to gently tear the cuticle without rupturing the gut tissue, releasing hemolymph into the oil. The ${\text{NH}}_{4}^{+}$ levels of the collected hemolymph droplets were measured as free ion activities using ion-selective microelectrodes (ISMEs). The construction of microelectrodes has been previously described in detail (Donini and O'Donnell, 2005). The following ionophore cocktails (Fluka, Buchs, Switzerland) and back-fill solutions (in parentheses) were used: ${\text{NH}}_{4}^{+}$ Ionophore I Cocktail A (100 mmol l^−1^ NH_4_Cl). The ISMEs were calibrated in the following solutions (mmol l^−1^): ${\text{NH}}_{4}^{+}$; (0.1, 1, 10) NH_4_Cl. Voltages were recorded and analyzed in LabChart 6 Pro software (AD Instruments Inc.).

### Scanning ion-selective microelectrode technique (SIET)

The scanning ion-selective electrode technique (SIET) system used in this study to measure ${\text{NH}}_{4}^{+}$ flux from the anal papillae of larvae has been previously described (Chasiotis et al., 2016). Larvae were mounted in a Petri dish using beeswax, leaving the anal papillae exposed and immobilized for measurements. Voltage gradients over an excursion distance of 100 μm were recorded adjacent to the papillae with a ${\text{NH}}_{4}^{+}$ microelectrode (see ISME) in a 4 ml bath of 0.5 mmol l^−1^ NH_4_Cl in double distilled water (ddH_2_O). The sampling protocol utilized here was outlined elsewhere (Chasiotis et al., 2016). Readings were taken along the middle to distal portion of the anal papillae at five equally spaced sites. Background voltage gradients were taken 3,000 μm away from the anal papillae using the same sampling protocol and were subtracted from the voltage gradients recorded at the papillae. To calculate ${\text{NH}}_{4}^{+}$ flux, voltage gradients were used to calculate ${\text{NH}}_{4}^{+}$ concentration gradients using the following formula:

$$\begin{array}{l} \Delta C\;=C_{B}\;x\;{\text{10}}^{\left(\Delta V/S\right)}-C_{B}, \end{array}$$

where Δ*C* is the concentration gradient (μmol l^−1^ cm^−3^), *C*_*B*_is the background concentration of ${\text{NH}}_{4}^{+}$ in the bath solution, Δ*V* is the voltage difference between the two points, and *S* is the slope of the electrode (the voltage difference for a 10-fold difference in ${\text{NH}}_{4}^{+}$ concentration). The ${\text{NH}}_{4}^{+}$ flux was then calculated using the concentration gradient (Δ*C)* and Fick's law of diffusion:

$$\begin{array}{l} J\;=D\left(\Delta C\right)/\Delta X, \end{array}$$

where *J* is the net flux of the ${\text{NH}}_{4}^{+}$ (pmol cm^−2^ s^−1^), *D* is the diffusion coefficient of ${\text{NH}}_{4}^{+}$ (2.09 x 10^−5^ cm^2^ s^−1^), Δ*C* is the concentration gradient calculated above, and Δ*X* is the excursion distance used to measure the voltage gradients (here 0.01 cm). The ${\text{NH}}_{4}^{+}$ fluxes were recorded from larvae treated with β-lac dsRNA (control), AeAmt2 dsRNA, or a combination of AeAmt1, AeAmt2, AeRh50-1, and AeRh50-2 dsRNA, where specified.

### Statistical analysis and phylogeny

Statistical analyses in this study were computed using Prism® 7.00 (GraphPad Software, La Jolla, CA, USA). An unpaired two-tailed *t*-test was performed for comparisons between two groups, unless specified. For comparisons of multiple groups for a single treatment (qRT-PCR studies), a one-way ANOVA was performed using the Holm-Sidak method for multiple comparisons. The adjusted *P*-value accounting for multiple testing is indicated. For SIET data, a single biological replicate is defined as the average flux from 5 sites along a single papilla from a single larva.

An unrooted maximum likelihood tree based on the JTT matrix-based model was constructed in MEGA7 from multiple sequence alignment of 16 amino acid sequences using Clustal X (Kumar et al., 2016). The Protter protein visualization software was used to generate the transmembrane plot of AeAmt2 (Omasits et al., 2014).

## Results

### Characterization, expression, and localization of AeAmt2 in the anal papillae

**Figure 1:**
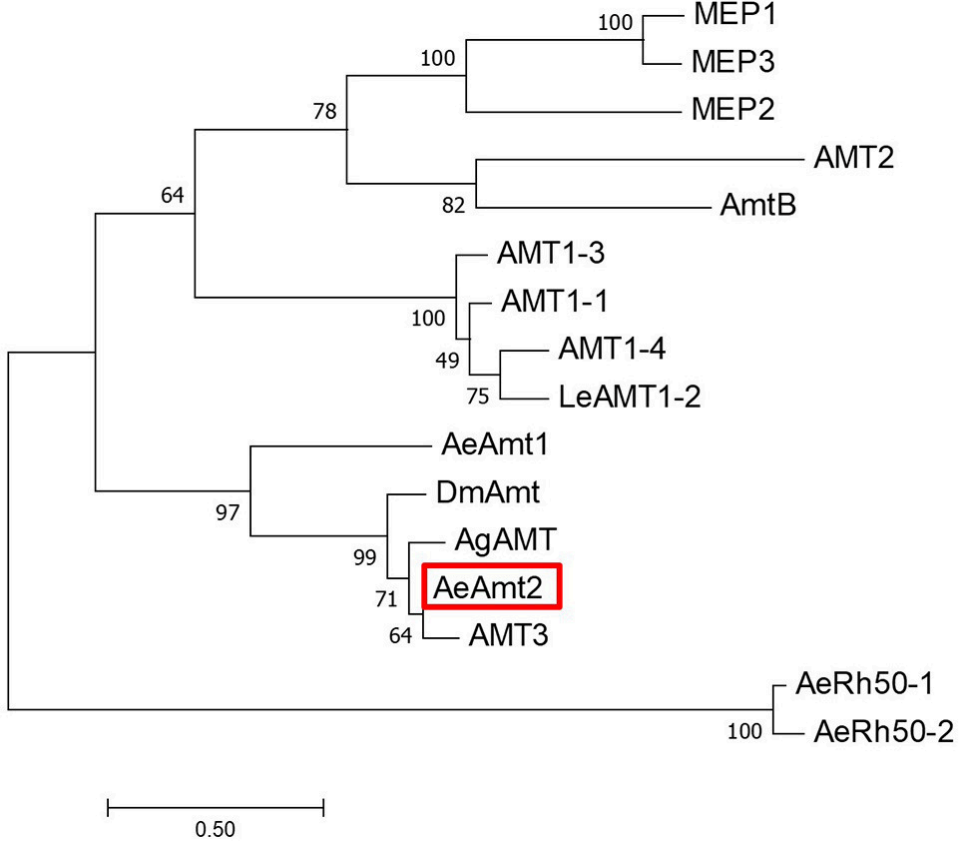
Unrooted maximum likelihood tree for 16 ammonium transporters (Amt), Rhesus glycoproteins (Rh), and methylammonium permeases (Mep). The percentage of trees in which the associated taxa clustered together from 500 replicates is shown next to the branches. The scale bar represents the number of amino acid substitutes per site, with tree branches drawn to scale. The boxed sequence is the subject of the current study. The analysis involved the multiple alignment of 16 full length amino acid sequences of 16 different genes using Clustal X prior to tree construction. Species name, gene, and GenBank accession number (in parentheses): *Saccharomyces cerevisiae* MEP1 (NP_011636), *Saccharomyces cerevisiae* MEP3 (EGA76487), *Saccharomyces cerevisiae* MEP2 (NP_011636), *Arabidopsis thaliana* AMT2 (NC_025010.1), *Escherichia coli* AmtB (Z71418.1), *Arabidopsis thaliana AMT 1-3* (NC_025010.1), *Arabidopsis thaliana* AMT 1-1 (NC_025010.1), *Arabidopsis thaliana* AMT 1-4 (NC_025010.1), *Lycopersicon esculentum* LeAMT1-2 (NC_025010.1), *Aedes aegypti AeAmt1* (XP_001652713.1), *Drosophila melanogaster* DmAmt (NP_001097800), *Anopheles gambiae* AgAMT (XM_318439), *Aedes aegypti* AeAmt2 (A0A1S4FGF9), *Aedes albopictus* AMT3 (XM_020076522), *Aedes aegypti* AeRh50-1 (AY926463.1), *Aedes aegypti* AeRh50-2 (AY926464.1).

**Figure 2:**
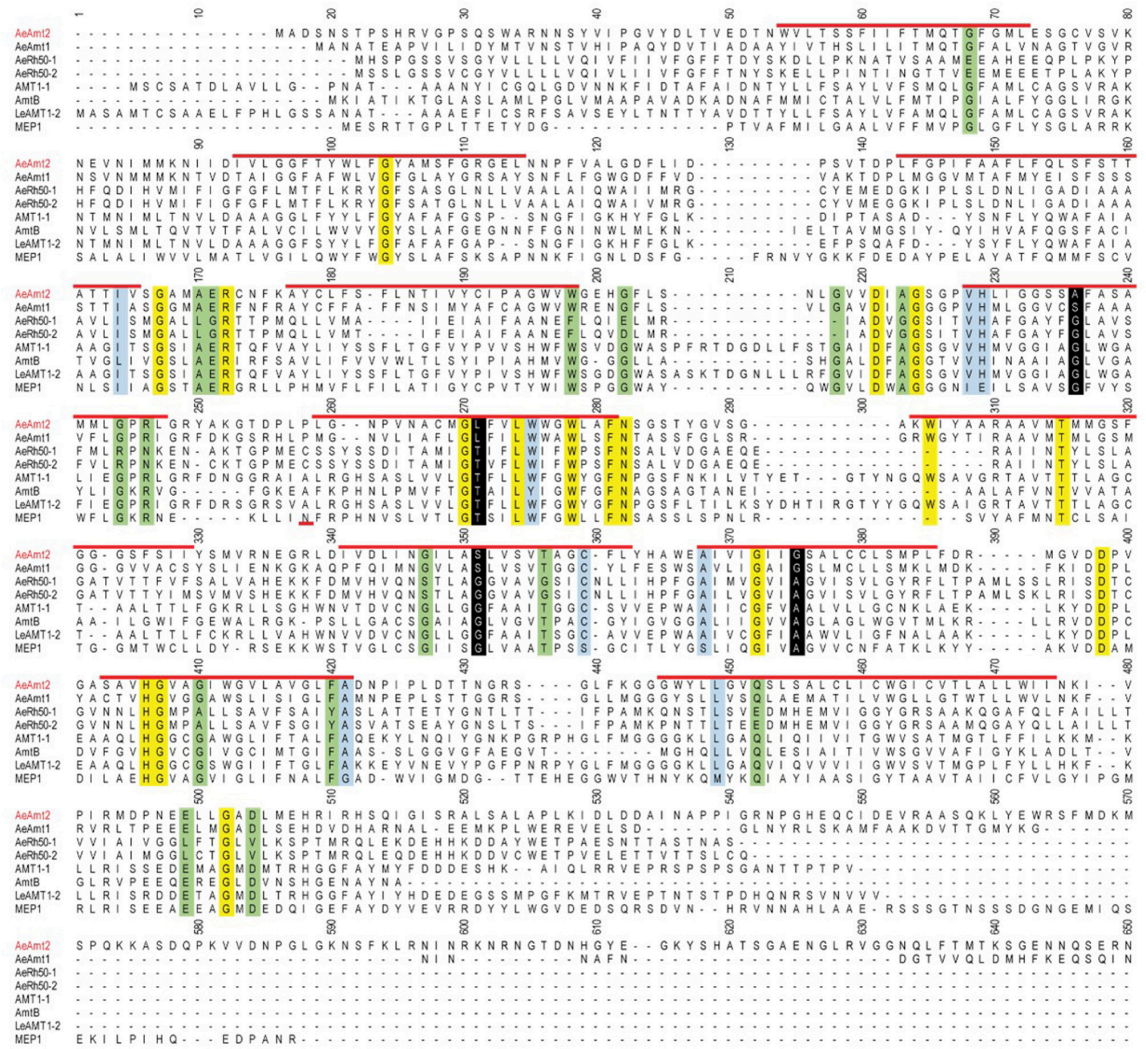
Amino acid sequence alignment of AeAmt2 and selected members of the Amt/MEP/Rh family. *A. aegypti* AeAmt2 is labeled in red, and predicted transmembrane helices from 1 to 11 beginning at residue 54 are indicated by the red lines (VectorBase). Sequence number according to LeAMT1-2 of *L. esculentum* is indicated, and 577 (out of 585 total) amino acid residues for AeAmt2 are shown. Highly conserved residues are highlighted in yellow, mostly conserved residues (7 out of 8 members have conserved amino acid residues) are highlighted in blue, highly conserved sequences in Amt/MEP proteins but not metazoan Rh proteins are highlighted in green, and conserved residues between AeAmt1 and AeAmt2 in *A. aegypti* that are different to the conserved residues in the other members are highlighted in black. Species names corresponding to amino acid sequences of ammonia transporters are as follows (in parentheses): AeAmt2 (*A. aegypti*), AeAmt1 (*A. aegypti*), AmtB (*E. coli*), AMT1-1 (*A. thaliana*), MEP1 (*S. cerevisiae*), Le-AMT1-2 (*L. esculentum*), AeRh50-1 (*A. aegypti*), and AeRh50-2 (*A. aegypti*). The alignment was constructed using the ClustalW algorithm.

**Figure 3:**
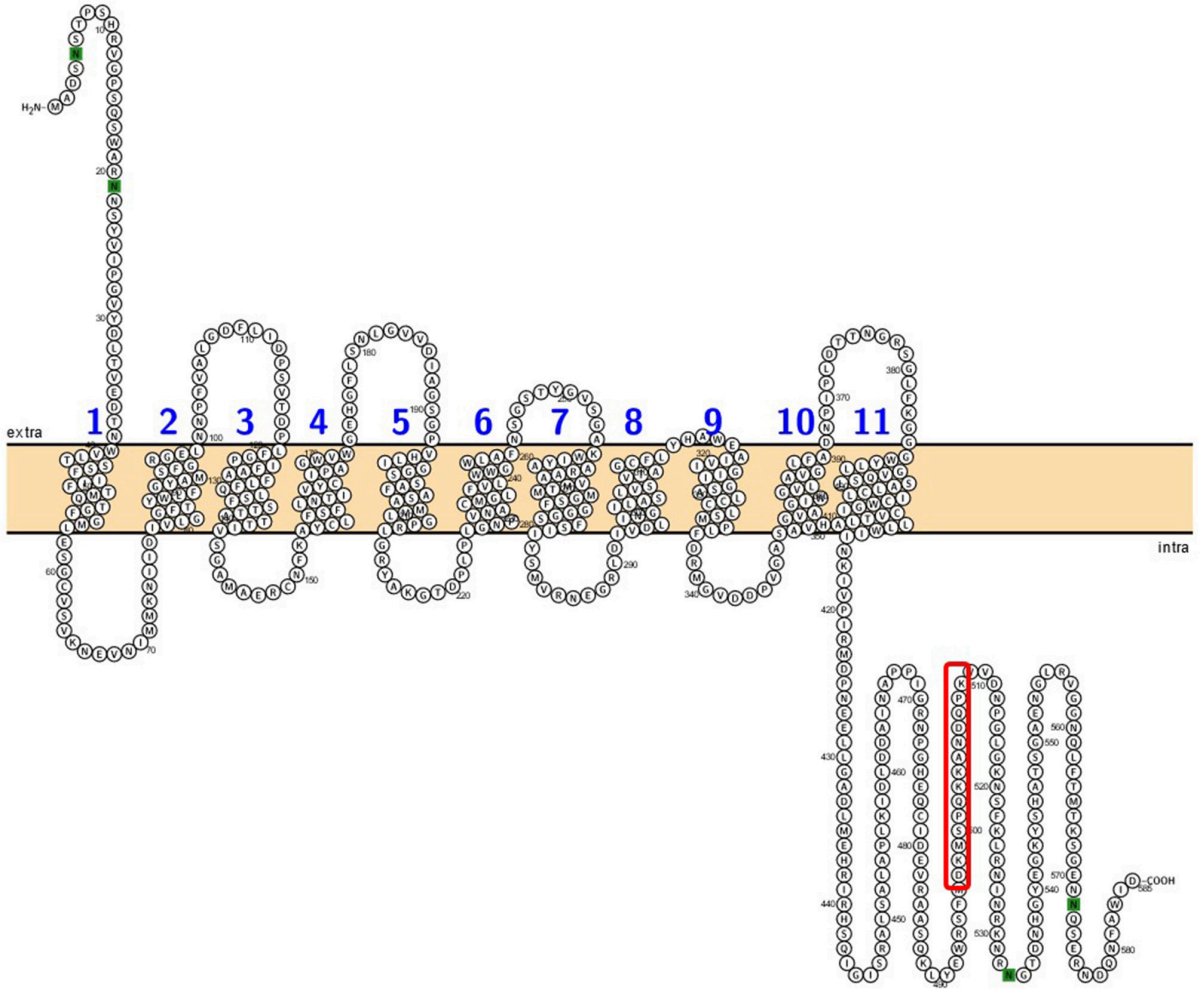
Amino acid plot of *Aedes aegypti* AeAmt2. The plot of predicted sequence features of AeAmt2 depicts 11 transmembrane domains, an extracellular (extra) N-terminus and an intracellular (intra) C-terminal region. Amino acids highlighted in green represent N-glycosylation sites. The amino acid sequence boxed in red represents the epitope used to generate the custom AeAmt2 antibody used in this study (see section Materials and Methods).

**Figure 4:**
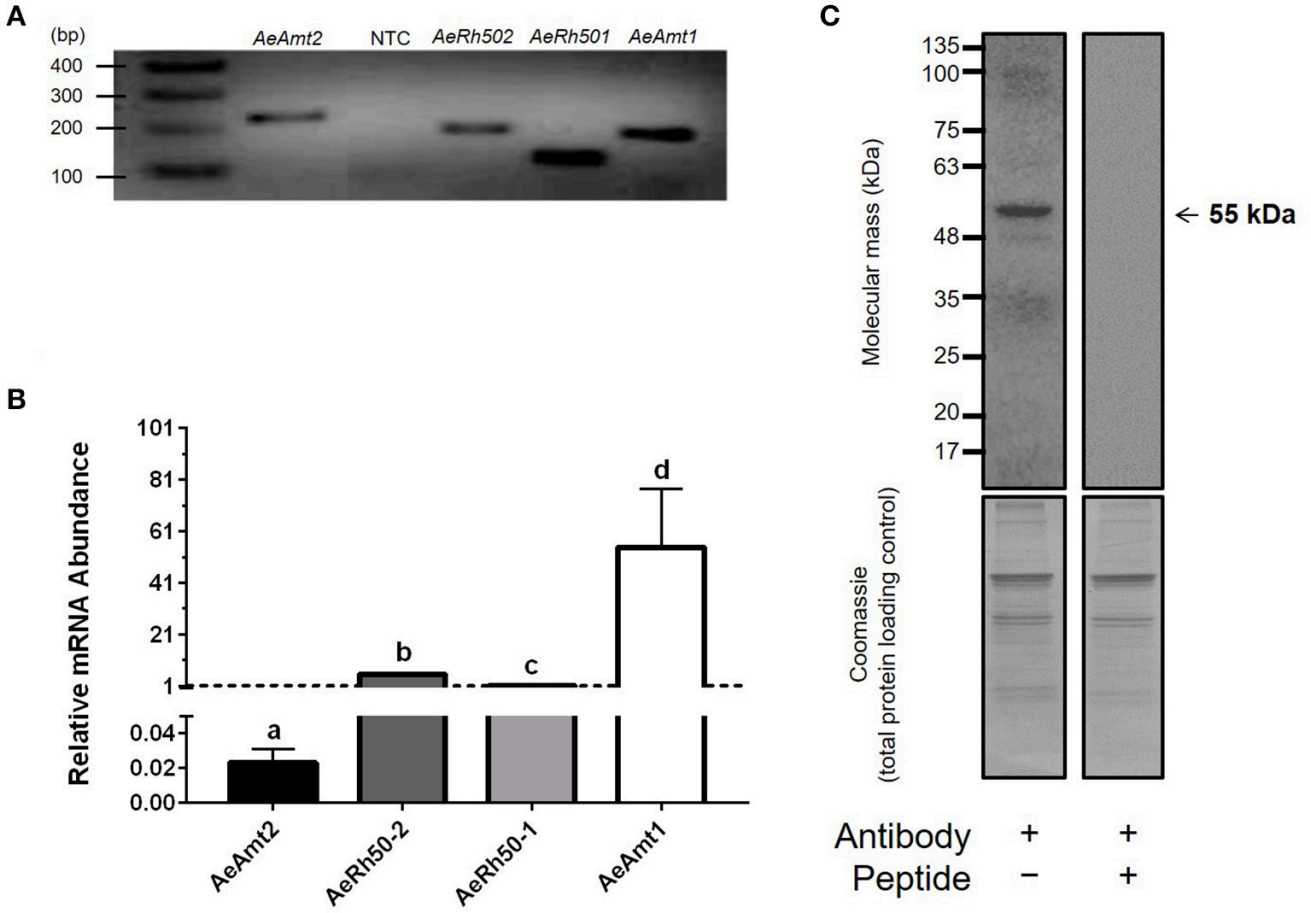
Ammonia transporter expression in the anal papillae of larval *A. aegypti*. **(A)** RT-PCR amplicon of *AeAmt2*, the corresponding no template control (NTC) for *AeAmt2* primers, *AeRh50-2, AeRh50-1*, and *AeAmt1* expression in the anal papillae resolved by gel electrophoresis. **(B)** Comparison of *AeAmt2, AeRh50-1, AeRh50-2*, and *AeAmt1* mRNA abundance in the anal papillae. Each gene was normalized to 18s ribosomal RNA abundance in the anal papillae and was expressed relative to *AeRh50-1* (assigned a value of 1). The dashed line illustrates *y* = 1. Data are expressed as mean values ± SEM (*n* = 3). Letters denote significant differences in relative mRNA abundance based on a One-way ANOVA (Holm-Sidak for multiple comparisons) of log transformed values (*p* = 0.00002). **(C)** Representative Western blot of larval anal papillae homogenates probed with AeAmt2 antisera revealing a single putative monomer at ~55 kDa (top). The single 55 kDa band was blocked by antibody pre-absorption with the immunogenic peptide. Coomassie total protein staining, used as a loading control, is shown in the lower.

*AeAmt2* is a member of the Amt family of ammonia transporters, sharing significant homology with putative or confirmed ammonia transporter proteins from other insects and to a lesser extent, Amts from plants (Figure 1). Multiple amino acid sequence alignment of eight members of the Amt/MEP/Rh family illustrates that the highly conserved residues in this family are also conserved in AeAmt2 (Figure 2). The transcript of *AeAmt2* is predicted to encode a peptide of 585 amino acid residues with a predicted molecular mass of 63.31 kDa (VectorBase) comprising 11 transmembrane helices and an extracellular N-terminus and intracellular C-terminus (Figure 3). To determine whether *AeAmt2* was expressed in the anal papillae, RT-PCR and agarose gel electrophoresis was performed (Figure 4A). A single distinct product for *AeAmt2* was observed at 220 base pairs. qRT-PCR was then used to examine relative *AeAmt2* transcript abundance in the anal papillae along with *AeAmt1, AeRh50-1*, and *AeRh50-2* (Figure 4B). *AeAmt2* mRNA abundance in the anal papillae was significantly lower in comparison to all other ammonia transporter mRNA examined, being ~50 and ~2,500 times less abundant than *AeRh50-1* and AeAmt1 within this organ, respectively. Western blot analysis in combination with a peptide block revealed one specific, putative AeAmt2 protein band in the anal papillae that does not appear when the antibody is preabsorbed with immunogenic peptide (Figure 4C). This putative AeAmt2 is 55 kDa in molecular mass. For densitometric analysis of AeAmt2 for all experiments, the 55 kDa band was used for protein quantification.

**Figure 5:**
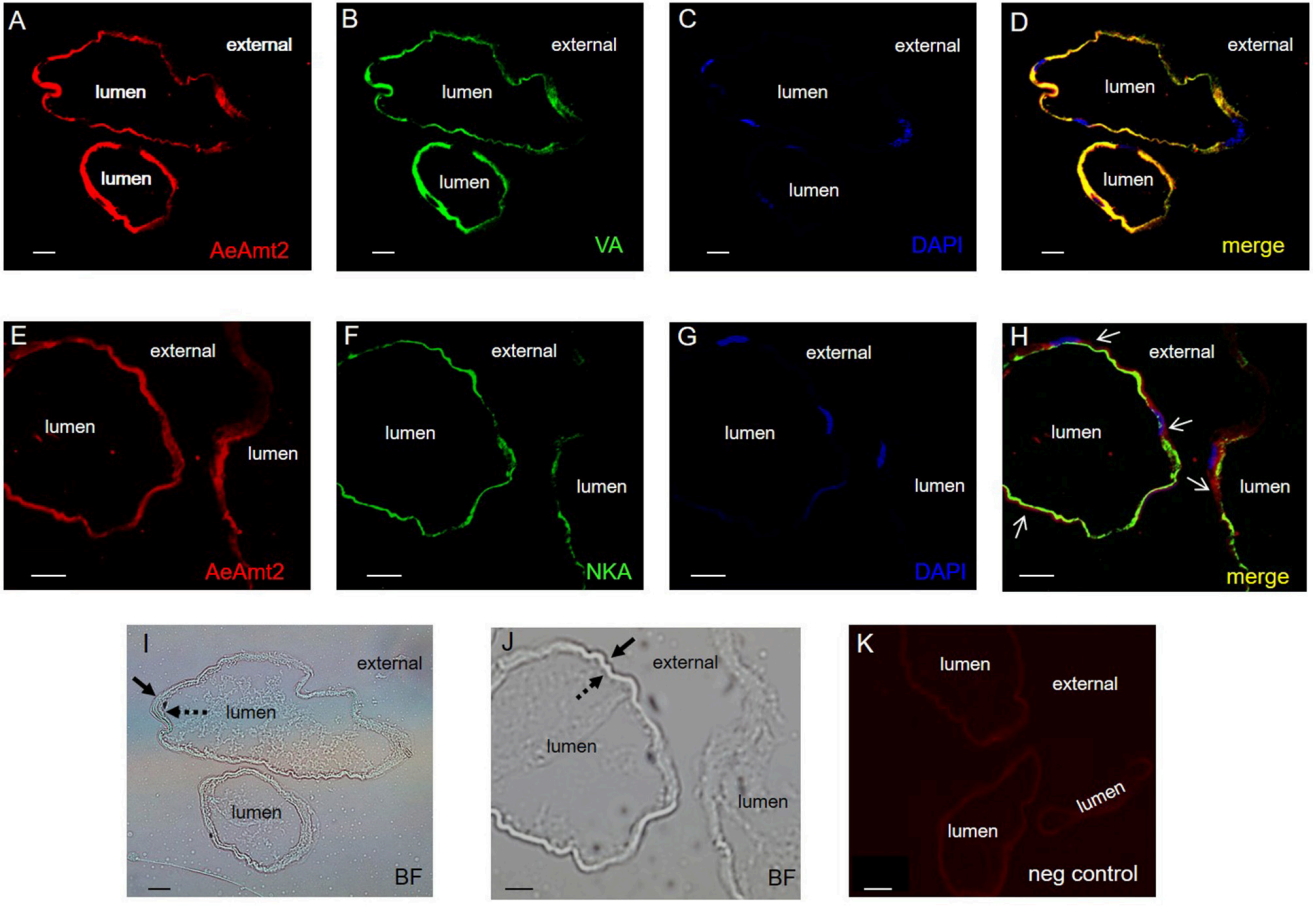
Immunolocalization of AeAmt2 in the anal papillae of *A. aegypti* larvae. Representative paraffin-embedded cross sections of anal papillae showing immunoreactivity for: **(A,E)** AeAmt2 (red), **(B)** the V_0_ subunit of V-type H^+^ ATPase (VA, green), **(F)** Na^+^-K^+^-ATPase (NKA, green), and **(C,G)** nuclei staining with DAPI (blue). **(D)** Merged image of AeAmt2, VA, and DAPI, and **(H)** merged image of AeAmt2, NKA, and DAPI. White arrows indicate areas with nuclei, where separation of the apical and basolateral membranes is observed. Cross sections of anal papillae in brightfield (BF) corresponding to **(I)** panels **(A–D)** and **(J)** panels **(E–H)**, demonstrating the apical (solid arrows) and basolateral (dashed arrows). **(K)** Control cross section of anal papillae (primary antibodies omitted). Scale bars **(A–K)**: 20 μM.

The ion-motive pumps V-type H^+^-ATPase (VA) and Na^+^/K^+^-ATPase (NKA) have been localized to the apical and basolateral membranes, respectively, within the anal papillae epithelium (Patrick et al., 2006). In paraffin-embedded cross sections of anal papillae from fourth instar larvae reared in deH_2_O, AeAmt2 immunostaining (Figure 5A), apical VA immunostaining (Figure 5B) co-immunolocalize when the images are merged, indicated by the yellow fluorescence (Figure 5D). Nuclei staining with DAPI (blue) is visible in anal papillae cross sections (Figures 5C,D). Conversely, AeAmt2 (Figure 5E) and NKA immunostaining (Figure 5F) do not appear to co-immunolocalize when merged (Figure 5H). This is most clearly visible at regions surrounding the nuclei (Figures 5G,H) indicated by the white arrows, showing separation of basolateral NKA staining and AeAmt2 staining. Yellow fluorescence was observed irregularly in merged images of NKA and AeAmt2 staining, which we believe is the result of membrane folding due to tissue sectioning. Cross sections of anal papillae in brightfield corresponding to AeAmt2, VA, and NKA immunostained cross sections (panels A–D and E–H) are shown (Figures 5I,J). No immunofluorescence was observed in negative control slides examined at the same exposure as experimental slides (Figure 5K).

### AeAmt2 dsRNA knockdown in the anal papillae and ${\text{NH}}_{4}^{+}$ flux

**Figure 6:**
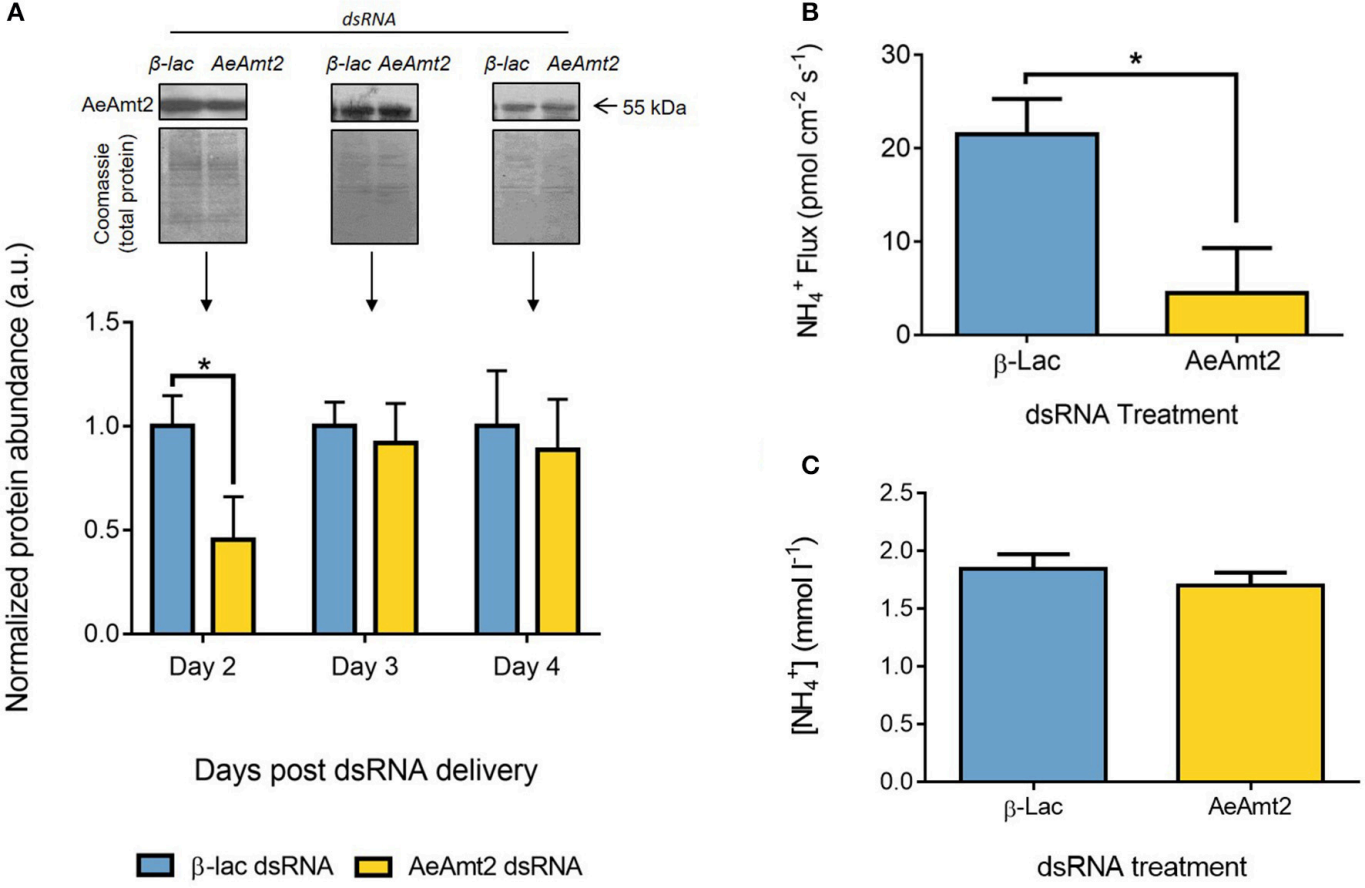
Effects of *AeAmt2* dsRNA treatment on AeAmt2 abundance in the anal papillae, ${\text{NH}}_{4}^{+}$ excretion from the anal papillae, and ${\text{NH}}_{4}^{+}$ concentration in the hemolymph. **(A)** Representative Western blots (1 representative of 3 replicates) and densitometric analysis of AeAmt2 (55 kDa) in the anal papillae on days 2–4 following dsRNA treatment. Each group was normalized to Coomassie total protein staining, used as a loading control, and is expressed relative to the β*-lac* control group (assigned a value of 1, *n* = 3). Asterisk denotes a significant difference in normalized protein expression compared to the β*-lac* control group based on an unpaired one-tailed *t*-test of relative density (*p* = 0.0491). **(B)** Scanning ion-selective electrode technique (SIET) measurements of ${\text{NH}}_{4}^{+}$ flux across the anal papillae epithelium 2 days post β*-lac* and *AeAmt2* dsRNA treatment (*n* = 10). Asterisk denotes a significant difference in ${\text{NH}}_{4}^{+}$ efflux compared to the β*-lac* control group based on an unpaired *t*-test (*p* = 0.0125). **(C)** Ion-selective microelectrode measurements of ${\text{NH}}_{4}^{+}$ concentration in the hemolymph of larvae at 2 days post β*-lac* and *AeAmt2* dsRNA treatment (*n* = 10). Data is shown as mean values ± SEM.

**Figure 7:**
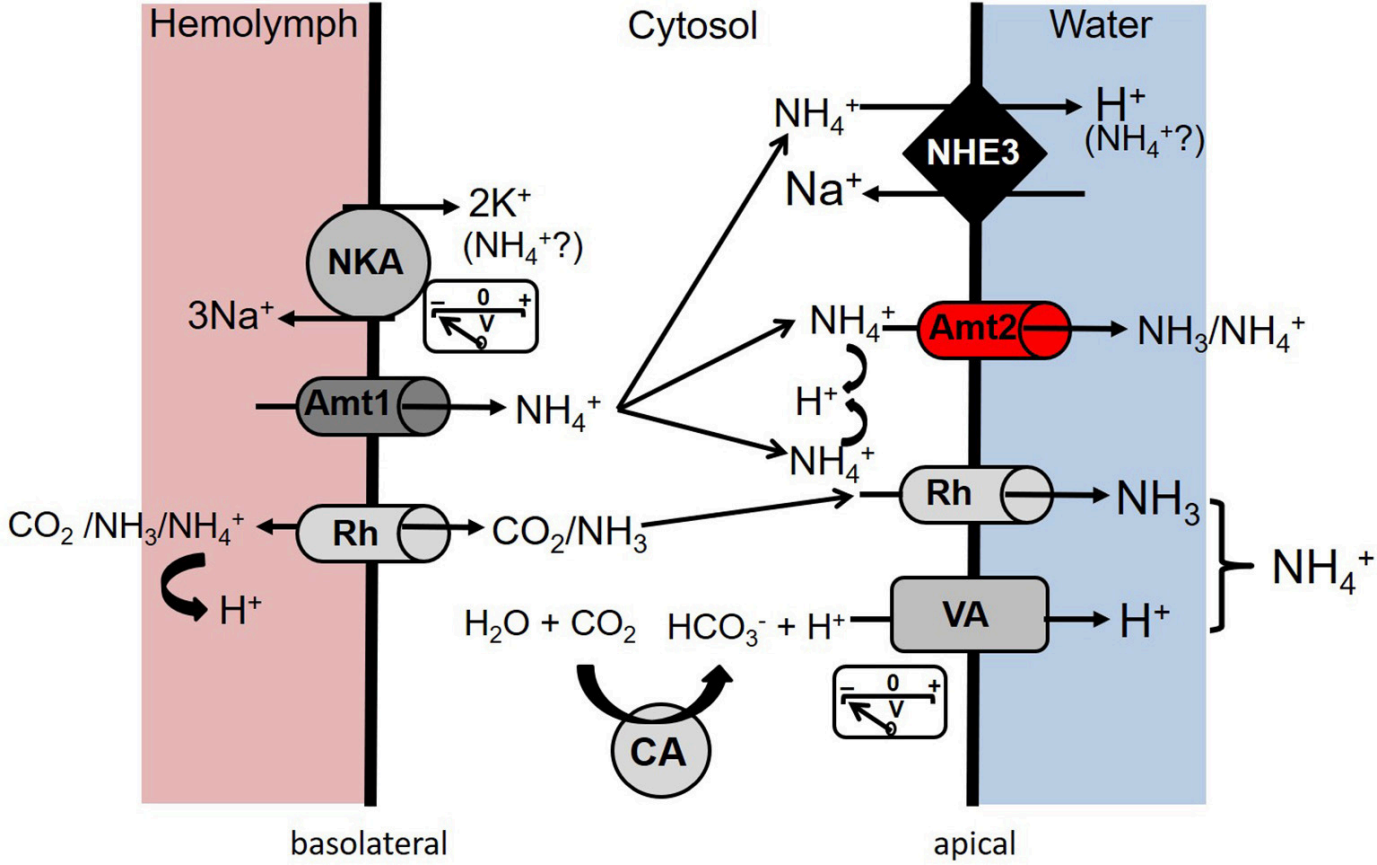
Current model of putative transcellular ammonia (NH_3_/${\text{NH}}_{4}^{+}$) transport mechanisms in the anal papillae epithelium of *A. aegypti* larvae in freshwater conditions (adapted from Chasiotis et al., 2016 copyright The Company of Biologists). Basolateral NKA provides a cytosol negative voltage potential that could serve to drive ${\text{NH}}_{4}^{+}$ from the hemolymph to the cytosol through AeAmt1 (Amt1). Partial pressure gradient (ΔP_NH3/CO2_) driven entry of CO_2_ and NH_3_ into the cytosol occurs through one of the two Rh proteins (AeRh50-1 or AeRh50-2). On the apical side, AeAmt2 facilitates NH_3_/${\text{NH}}_{4}^{+}$ exit from the cytosol to the aqueous habitat either through ${\text{NH}}_{4}^{+}$ transport, NH_3_ transport following ${\text{NH}}_{4}^{+}$ recruitment and deprotonation, or NH_3_/H^+^ co-transport. ${\text{NH}}_{4}^{+}$ in the cytosol may exit from the apical side to the aqueous habitat through the sodium-hydrogen exchanger 3 (NHE3) in exchange for Na^+^. An Rh protein (Rh) on the apical side may function to excrete NH_3_ through an ammonia-trapping mechanism exploiting an acidified boundary layer generated by apical VA and partly by NHE3. Cytoplasmic carbonic anhydrase (CA) contributes to the cytosol negative voltage potential and the maintenance of the acidified boundary layer by supplying H^+^ for VA.

AeAmt2 protein abundance in the anal papillae was significantly reduced by dsRNA knockdown 2 days post treatment and returned to control levels thereafter (Figure 6A). As a result, functional analyses in response to AeAmt2 protein knockdown was performed 2 days post dsRNA treatment. ${\text{NH}}_{4}^{+}$ efflux from the anal papillae was significantly reduced by 3-fold at 2 days post *AeAmt2* dsRNA treatment (Figure 6B), but ${\text{NH}}_{4}^{+}$ hemolymph levels were unchanged in response to dsRNA-mediated AeAmt2 knockdown in the anal papillae (Figure 6C). A summarized model of the current mechanisms of ammonia excretion in the anal papillae, reflecting the contribution of AeAmt2 in facilitating this process is provided (Figure 7).

### Effects of HEA on ammonia transporter expression within the anal papillae

**Figure 8:**
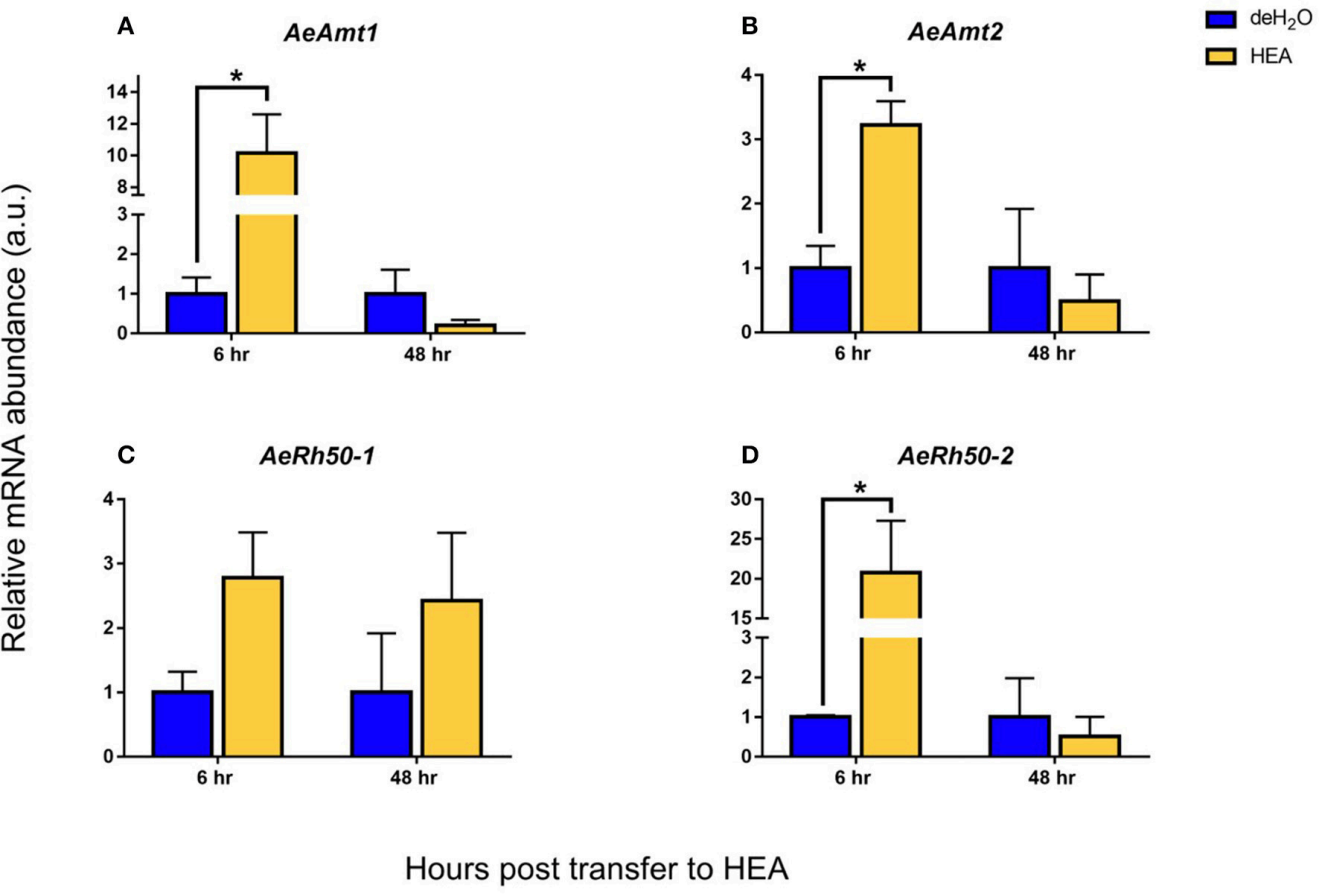
Effects of high environmental ammonia (HEA) on transcript abundance of ammonia transporter genes in the anal papillae of *A. aegypti* larvae. Relative mRNA abundance of **(A)** *AeAmt1*, **(B)** *AeAmt2*, **(C)** *AeRh50-1*, and **(D)** *AeRh50-2* at 6 and 48 h post transfer to dechlorinated tap water (deH_2_O) or HEA (5mM NH_4_Cl) (*n* = 3). Each gene was normalized to 18s ribosomal RNA abundance and expressed relative to levels in the control deH_2_O group (assigned a value of 1). Data is shown as mean values ± SEM. Asterisks denote statistically significant differences between deH_2_O and HEA groups (Unpaired *t*-test, *p* ≤ 0.05).

**Figure 9:**
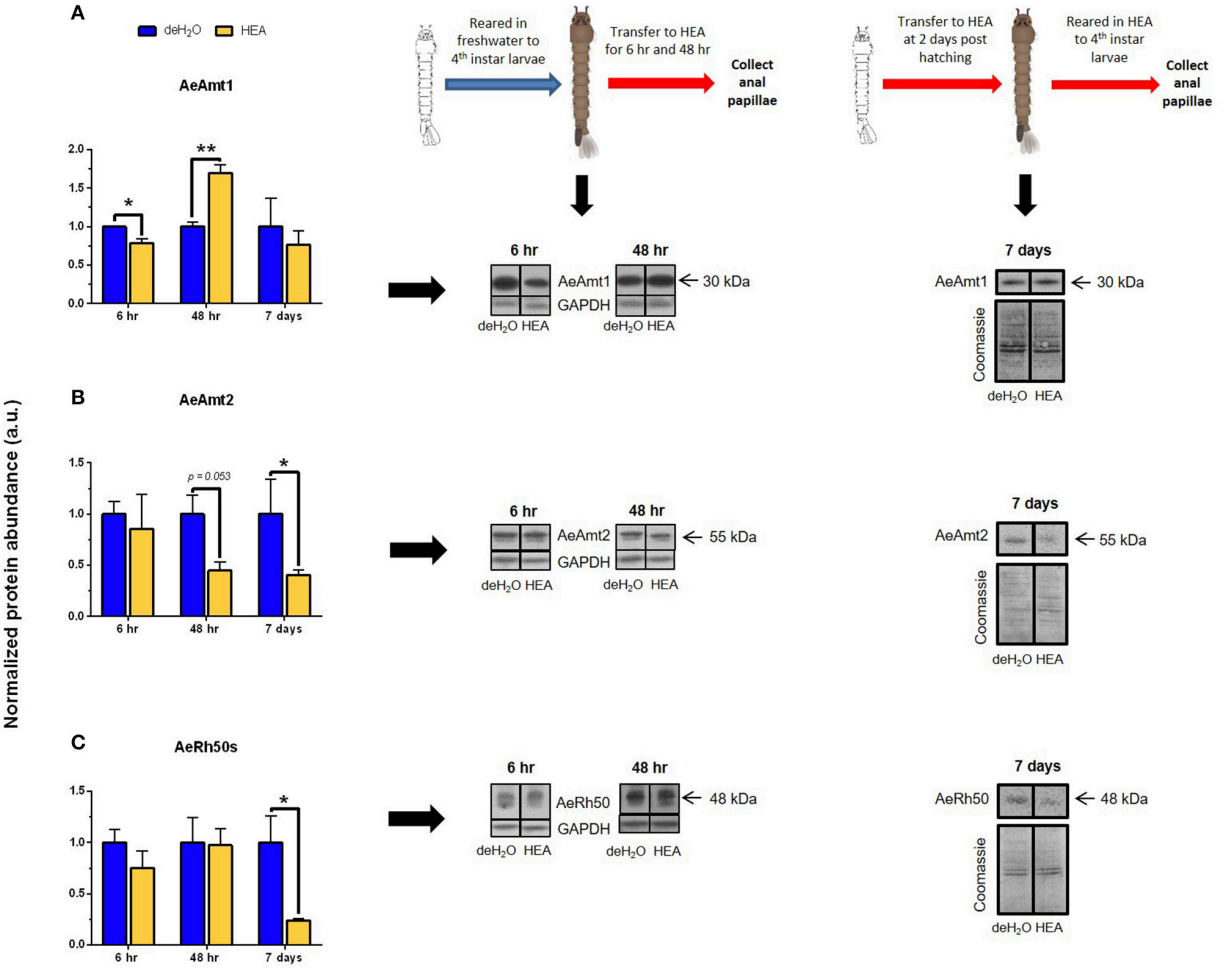
Effects of high environmental ammonia (HEA) exposure on protein abundance of ammonia transporter genes in the anal papillae of fourth instar *A. aegypti* larvae. **(A)** AeAmt1 normalized monomer (30 kDa) abundance, **(B)** AeAmt2 normalized monomer (55 kDa) abundance, and **(C)** AeRh50s normalized monomer (48 kDa) abundance, and corresponding representative Western blots (right panels, 1 representative of three replicates) at 6, 48 h, and 7 days in dechlorinated tap water (deH_2_O) or HEA (5 mM NH_4_Cl) (*n* = 3). For 6 and 48 h HEA exposure, protein abundance was normalized to GAPDH abundance in the anal papillae. For 7-day HEA exposure, protein abundance was normalized to total protein (Coomassie). Normalized protein abundance for each protein is expressed relative to the control deH_2_O group (assigned a value of 1). Data is shown as mean values ± SEM. Asterisks denote statistically significant differences between deH_2_O and HEA groups ($$unpaired *t*-test, ^*^p ≤ 0.05, ^**^p ≤ 0.005).

The acute (6 h), semi-chronic (48 h), and chronic (7 days) changes that occur in ammonia transporter transcript and protein abundance in the anal papillae in response to HEA (5 mM NH_4_Cl) were examined (Figures 8, 9). Significant alterations to both mRNA and protein abundance were evident but did not always follow the same trends. The mRNA abundance of *AeAmt1, AeAmt2*, and *AeRh50-2* followed the same trend in response to HEA; however, the abundance of their respective protein did not. After larvae were exposed to HEA for 6 h, the mRNA abundance of *AeAmt1, AeAmt2*, and *AeRh50-2* in anal papillae was 10, 3, and 20-fold higher than that of controls (Figure 8). The protein abundance of AeAmt1 in anal papillae was significantly lower after 6 h larval exposure to HEA compared to controls (Figure 9A), whereas there was no difference in the protein abundance of AeAmt2 or the AeRh50s after 6 h (Figures 9B,C). When larvae were exposed for 48 h to HEA there was no difference in mRNA abundance of any of the ammonia transporter genes in the anal papillae compared to controls (Figures 8A–C); however, the protein abundance of AeAmt1 was significantly higher (Figure 9A). When larvae were reared for 7 days in HEA (5 mM NH_4_Cl), AeAmt1 protein abundance did not change (Figure 9A), however, significant decreases in AeAmt2 and AeRh50s protein abundance by 60 and 77%, respectively, was observed (Figures 9B,C).

## Discussion

### A novel putative ammonia transporter of *Aedes aegypti*, AeAmt2

In the present study we have identified and characterized a novel putative ammonia transporter, *AeAmt2*, in the mosquito *A. aegypti*. This protein aligns with members of the family of ammonium transporters (Amt/MEP/Rh) from plants, bacteria, fungi, and vertebrates and shares high sequence homology with antennal-expressed AgAMT (Pitts et al., 2014) of the mosquito *A. gambiae*, the antennal-expressed DmAmt from the fruitfly *D. melanogaster* (Menuz et al., 2014), and an uncharacterized AMT3 in the mosquito *A. albopictus. A. aegypti* AeAmt2 and other insect Amts cluster with some electrogenic plant Amts (e.g., LeAMT1;2) in comparison to electroneutral bacterial Amts (e.g., AmtB) (see Figure 1).

Similar to other Amt proteins, AeAmt2 is comprised of 11 transmembrane helices which vary in length (19-30 amino acids), and possesses the common extracellular N-terminus and intracellular C-terminus topology conserved in this family (Severi et al., 2007). The cytoplasmic C-terminus of AeAmt2 is notably longer in comparison to other members of the Amt/MEP/Rh family (Figures 2, 3). Multiple sequence alignment of amino acid residues from *A. aegypti* Amt and Rh proteins, specifically AeAmt2, with other Amt/MEP proteins from plants, fungi, and bacteria demonstrate the presence of highly conserved residues which cluster around the narrow hydrophobic pore formed by the monomer (Khademi et al., 2004; Zheng et al., 2004). Similar to the bacterial AmtB, two highly conserved histidine residues (H229 and H406) are located at the N-termini of pseudo-symmetric transmembrane helices 5 and 10 of the AeAmt2 monomer (Zheng et al., 2004). The imidazole side chains of H229 and H406 and the acidic side chains of the preceding highly conserved aspartates (D221 and D398) play a role in hydrogen bonding and structural support, respectively (Zheng et al., 2004). Two highly conserved threonine residues (T315 and T356) among the Amt/MEP/Rh family which are present in AeAmt2 were suggested to be functionally relevant, contributing to a tightly-packed pore surface on the periplasmic side of the monomer (Khademi et al., 2004; Zheng et al., 2004). Furthermore, mutation of a highly conserved tryptophan 148 residue (W198 in the present study) at the periplasmic entry of the hydrophobic pore of each monomer in Amt/MEP proteins, also conserved in transmembrane helix four of AeAmt2, demonstrated that the substrate of the channel is an ion and W148 is important for restricting the conductance and increasing selectivity of AmtB to ${\text{NH}}_{4}^{+}$ (Fong et al., 2007). Together, this structural information suggests that AeAmt2 possesses all the ammonia transporting capabilities of other known ammonia transporters.

### Expression of AeAmt2 in the larval anal papillae

The anal papillae of *A. aegypti* larvae are important organs for the excretion of ammonia directly into the aquatic environment (Donini and O'Donnell, 2005). The syncytial epithelium of the anal papillae expresses the ammonia transporters, *AeAmt1, AeRh50-1*, and *AeRh50-2* and knockdown experiments revealed their importance in ammonia transport by the anal papillae (Chasiotis et al., 2016; Durant et al., 2017). We detected *AeAmt2* mRNA in the anal papillae but the transcript abundance of *AeAmt2* was significantly lower than that of the other ammonia transporters (Figure 4). An antibody generated against a unique sequence in the predicted cytosolic region of the AeAmt2 protein detected a 55 kDa signal in western blots which was abolished by pre-incubation of the antisera with immunogenic peptide. Occasionally, 48 and 63 kDa signals also appeared on blots (not shown). The 63 kDa signal corresponds to the predicted mass of the full-length sequence of the AeAmt2; however, since this only occasionally appeared while the 55 kDa band consistently appeared, we suggest that AeAmt2 may undergo post-translational processing. For example, the mature, functional bacterial AmtB protein is derived from cleavage of a signal sequence from the preprotein (Thornton et al., 2006). The dichotomy of relatively low *AeAmt2* mRNA levels but high protein abundance that we observed could simply be a result of low protein turnover, discussed below.

AMT/Mep/Rh protein monomers are known to trimerize in the membrane when functional (Khademi et al., 2004; Zheng et al., 2004; Gruswitz et al., 2010). We did not see the trimeric form in western blots likely because of the denaturing conditions used, separating the trimeric forms into respective monomers (Severi et al., 2007). The significance of the trimeric arrangement of Amt/MEP/Rh proteins is attributed to interactions with the trimeric regulatory P_II_ protein, GlnB or its homolog GlnK in prokaryotes, with each monomer of the Amt/MEP/Rh trimer conducting substrate independently (Coutts et al., 2002; Conroy et al., 2007; Gruswitz et al., 2007). The regulatory P_II_ proteins are stable trimers with extended T-loops protruding from each monomer (Andrade and Einsle, 2007). In *Azotobacter vinelandii*, evidence of a direct association of GlnK with the cytoplasmic face of Amt-1 in response to high levels of ammonium in the cell was provided (Coutts et al., 2002), and physical obstruction of the pores of each monomer of AmtB from *E. coli* through the insertion of the T-loops of the trimeric GlnK deep into each pore was shown (Conroy et al., 2007; Gruswitz et al., 2007). Furthermore, the regulation of Amt proteins in plants through post-translational modifications may provide insight into the mechanisms of regulation for invertebrate Amts which is presently unknown. *A. thaliana* AtAmt1;2 activity is regulated by phosphorylation, whereby dephosphorylation of all monomers comprising the trimer is essential for activation of ammonia transport with each monomer functioning independently (Neuhäuser et al., 2007).

Studies on mutant variants at the conserved cytoplasmic C-terminus region of AmtB proceeding transmembrane helix 11 in *E. coli* may imply an important functional or structural role at this region (Severi et al., 2007). Mutants were either inhibited in their ammonia transporting capabilities or were completely void of activity altogether, suggesting a gating mechanism of the trimer through the carboxyl tail of a single monomer. Mutation of a single glycine residue (G502 in this study, Figure 2) in the cytoplasmic C-terminus region, conserved in AeAmt2, within a single monomer led to cross-inhibition of ${\text{NH}}_{4}^{+}$ transport within the entire trimer in some plant Amts (Ludewig et al., 2003; Neuhäuser et al., 2007). The extensive cytoplasmic C-terminus of AeAmt2 in comparison to that of other members, possessing two putative N-glycosylation sites (Figure 3) and approximately 14 putative phosphorylation sites (not shown; Blom et al., 2004), may suggest a more complex role of this region. Regulation of AeAmt2 protein by any of the mechanisms described in other members of the Amt/MEP/Rh family outlined above are quite possible and require further investigation.

Immunohistochemistry on cross sections of anal papillae revealed that AeAmt2 is localized to the apical, water facing membrane of the epithelium. This is only the second cellular localization of an Amt protein in a functioning organ of an animal, and the first apical localization of an Amt protein in animals. An apical localization of AeAmt2 was unsurprising based on the distribution of Rh proteins in the anal papillae of *A. aegypti* larvae and the cortex cells of the rat kidney to opposite membranes (Quentin, 2003; Durant et al., 2017).

### Functional consequences of AeAmt2 protein knockdown in the anal papillae of larvae

RNA interference techniques in combination with SIET were used to elucidate an ammonia-transporting function of AeAmt2 directly at the anal papillae. dsRNA-mediated knockdown of AeAmt2 protein, verified in anal papillae to be ~50% at 2 days post dsRNA treatment resulted in a significant decrease in ${\text{NH}}_{4}^{+}$ efflux from the anal papillae, indicating that AeAmt2 is functioning to facilitate ammonia excretion within this organ. The current working model of transcellular ammonia excretion in the syncytial anal papillae epithelium has been updated to reflect the contribution of AeAmt2 (Figure 7) (Chasiotis et al., 2016). On the apical side of the epithelium, AeAmt2 likely facilitates ${\text{NH}}_{4}^{+}$ transport from the cytosol to the external surroundings for excretion by means of three possible mechanisms. Through electroneutral transport, an AeAmt2 trimer can recruit ${\text{NH}}_{4}^{+}$ from the cytosol where the proton is stripped and NH_3_ traverses the triple pore for excretion (Soupene et al., 1998; McDonald and Ward, 2016). By contrast, electrogenic transport of ${\text{NH}}_{4}^{+}$ at the apical membrane of papillae can occur through AeAmt2 function as a ${\text{NH}}_{4}^{+}$ uniporter, as a co-transporter for NH_3_/H^+^, or as an ${\text{NH}}_{4}^{+}$/H^+^ antiporter (Ludewig et al., 2002; Neuhäuser and Ludewig, 2014). The former two possibilities are unlikely due to the cytosolic negative potential of the anal papillae epithelium, driven largely by apical VA. A mechanism of ${\text{NH}}_{4}^{+}$/H^+^ antiport by AeAmt2 is more plausible, with VA providing the H^+^ gradient facilitating ${\text{NH}}_{4}^{+}$ exchange. In this manner, H^+^ is moved along its own electrochemical gradient allowing ${\text{NH}}_{4}^{+}$ to be moved against its electrochemical gradient.

The knockdown of AeAmt2 is assumed to be systemic but this did not have an effect on hemolymph ${\text{NH}}_{4}^{+}$ levels. Similarly, the knockdown of AeRh50-2 did not affect hemolymph ${\text{NH}}_{4}^{+}$ levels in larvae (Durant et al., 2017). In contrast, the knockdown of either AeAmt1 or AeRh50-1 results in alterations to hemolymph ${\text{NH}}_{4}^{+}$ levels whereby AeAmt1 knockdown causes an increase in ${\text{NH}}_{4}^{+}$, and AeRh50-1 knockdown results in decreased ${\text{NH}}_{4}^{+}$ levels and an acidification of the hemolymph (Chasiotis et al., 2016; Durant et al., 2017). Our interpretation of these results assumes a key role of the anal papillae in ammonia excretion; however, we do not propose that these are the only organs responsible for ammonia excretion. Decreasing AeAmt1 expression in papillae would decrease ammonia clearance from the hemolymph across the basal side of the papillae epithelia resulting in the observed increase of ${\text{NH}}_{4}^{+}$ hemolymph levels. In contrast, decreasing AeRh50-1 expression could lead to decreased hemolymph ${\text{NH}}_{4}^{+}$ levels if AeRh50-1 is on the basal side of the papillae epithelia because it could mediate back-flux of ammonia to the hemolymph when cytosolic levels become too high which may be expected since ammonia transport by AeAmt1 is energized by the activity of Na^+^/K^+^-ATPase (Chasiotis et al., 2016). The finding that hemolymph ${\text{NH}}_{4}^{+}$ levels are unaltered by AeAmt2 knockdown may suggest that disruption of apical pathways of ammonia excretion at the papillae are not as impactful to systemic hemolymph ammonia levels. This lends credence to the suggestion that AeRh50-2 may be expressed apically, as AeRh50-2 knockdown does not affect ${\text{NH}}_{4}^{+}$ hemolymph levels. Collectively, these results may also suggest that AeAmt1 function in the anal papillae is the most important for systemic regulation of ammonia levels with respect to the other three ammonia transporters expressed in the anal papillae.

### Ammonia transporter expression in response to HEA

In HEA conditions, larvae are faced with the challenge of combatting significant inwardly directed partial pressure (ΔP_NH3_) and ${\text{NH}}_{4}^{+}$ electrochemical gradients at the anal papillae and other osmoregulatory organs (Weihrauch et al., 2012b). Traditionally thought to be a passive process in aquatic animals, active ammonia excretion against a 4–8 fold inwardly directed ammonia gradient has been well documented in the gills of crabs (Weihrauch et al., 2004). We quantified the expression of AeAmts and AeRh50s at both the transcript and protein level in the anal papillae of larvae exposed to acute (6 h), semi-chronic (48 h) and long term (7 days) HEA exposure (5 mM NH_4_Cl). Significant increases in the mRNA abundance of both *AeAmts* as well as *AeRh50-2* in the anal papillae were observed after 6 h exposure to HEA (see Figure 8), as well as a decrease in AeAmt1 protein levels (see Figure 9). After 48 h in HEA, mRNA abundance of both AeAmts and both AeRh50s were no different than those in control larvae (Figure 8); however, an increase in basolateral AeAmt1 and a decrease in apical AeAmt2 protein abundance in the anal papillae was observed when compared with levels in control larvae (Figure 9). This result was similar to that found for a 7 day HEA exposure in that AeAmt2 protein abundance decreased relative to controls and AeAmt1 protein abundance remained unchanged (Figure 9). The finding that expression of a basolateral Amt (AeAmt1) is either unaffected or increases, and an apical Amt (AeAmt2) decreases simultaneously in response to HEA suggests a mechanism of ammonia clearance from the hemolymph whilst limiting ${\text{NH}}_{4}^{+}$ influx at the apical membrane. This may imply that AeAmt1 is the key driver of ammonia excretion in the anal papillae irrespective of external ${\text{NH}}_{4}^{+}$ levels. AeAmt1 function is likely driven by NKA activity (secondary active transport), as supported by pharmacological evidence (see Chasiotis et al., 2016) and would not be affected by changing NH_3_/${\text{NH}}_{4}^{+}$ gradients, whereas a decrease in apical transporter expression may limit NH_3_/${\text{NH}}_{4}^{+}$ influx into the anal papillae cytosol in HEA conditions. We were unable to discern specific changes in AeRh50-1 and AeRh50-2 protein abundance because the antibody detects both (Durant et al., 2017). Nevertheless, it may be reasoned that the increase in *AeRh50-2* mRNA abundance after 6 h in HEA led to changes in protein abundance of AeRh50-2.

Overall, the immediate and long term responses to HEA is reflective of the importance of these transporters in regulating ammonia excretion within the anal papillae against an inwardly directed gradient, where larvae have been shown to tolerate ${\text{NH}}_{4}^{+}$ hemolymph levels of between 1 and 3 mmol l^−1^ under normal freshwater conditions (Chasiotis et al., 2016; Durant et al., 2017). It is also very likely that due to post-translational modifications or conformational changes regulating Amt and Rh protein activity, turnover numbers of these proteins are limited even in HEA conditions. In this manner, changes in the activity of Amt and Rh proteins may be more indicative of their role in facilitating ammonia excretion in response to HEA.

In general, *A. aegypti* larvae appear to be more tolerant of high ammonia hemolymph levels and HEA in comparison to other aquatic invertebrates (Weihrauch et al., 2004; Weihrauch and Donnell, 2017). HEA levels of 5 mmol l^−1^ NH_4_Cl and above are lethal to the freshwater planarian, *Schmidtea mediterranea*, within 48 h (Weihrauch et al., 2012a). However, a significant increase in the mRNA of a Rh-like transporter upon exposure to 1 mmol l^−1^ for 48 h was reported. In the freshwater leech *Nephelopsis obscura*, increases in ammonia excretion within 1 h of exposure to HEA (1 mmol l^−1^ NH_4_Cl) was attributed to increases in metabolic rates due to stress, and mRNA levels of NoRhp, an Rh transporter, in the gills decreased after 7 days in HEA (Quijada-Rodriguez et al., 2015). Evidence that apical AeAmt2 and AeRh50 protein decreases in response to long term exposure to HEA may suggest a regulatory mechanism which prevents both chemical and/or electrochemical driven influx of ammonia at the anal papillae.

### Summary

The quality of larval habitats for egg hatching and development is a strong determinant of the selection of breeding sites for female *A. aegypti* (Chitolina et al., 2016). Vector control and surveillance programs are directly influenced by where mosquitoes breed (Barrera et al., 2008; Banerjee et al., 2015), highlighting the importance of understanding how *A. aegypti* can adapt to ammonia-rich raw sewage in septic tanks. Septic tank usage is common and widespread in tropical regions where *A. aegypti* are most prevalent which may explain the persistence of disease during dry periods or following control measures, such as removing artificial water containers (Burke et al., 2010). The aim of this study was to further our understanding of the molecular and physiological mechanisms of ammonia excretion in *A. aegypti* larvae attributing to their tolerance of HEA.

We have identified and characterized a novel ammonia transporter, *AeAmt2*, belonging to the Amt/MEP/Rh family of conserved ammonia transporters in bacteria, plants, and animals. This is only the second characterization and localization of an Amt protein in an animal. *AeAmt2* is expressed in the osmoregulatory anal papillae which are important organs for the excretion of ammonia directly from the hemolymph of *A. aegypti* larvae (Donini and O'Donnell, 2005). The apical localization of AeAmt2 in the anal papillae epithelium of *A. aegypti* larvae is the first apical localization of an Amt protein in animals. AeAmt2 plays a significant role in facilitating ammonia excretion from the anal papillae demonstrated by dsRNA-mediated knockdown studies using SIET. Furthermore, the involvement of AeAmt1, AeAmt2, AeRh50-1, and AeRh50-2 in facilitating ammonia excretion and the maintenance of ammonia hemolymph levels in response to HEA was investigated, providing insight into the tolerance of *A. aegypti* larvae to high ammonia. This is one of few comprehensive studies of the physiological mechanisms underlying ammonia excretion in *A. aegypti* larvae permitting them to survive and thrive in HEA habitats such as septic tanks.

## Author contributions

ACD and AD: designed the study; ACD: performed the experiments, data collection, data analysis, and wrote the manuscript; AD: secured funding and edited the manuscript.

### Conflict of interest statement

The authors declare that the research was conducted in the absence of any commercial or financial relationships that could be construed as a potential conflict of interest.
